# Metabolites Obtained from Boraginaceae Plants as Potential Cosmetic Ingredients—A Review

**DOI:** 10.3390/molecules29215088

**Published:** 2024-10-28

**Authors:** Ewelina Chrzanowska, Bożena Denisow, Halina Ekiert, Łukasz Pietrzyk

**Affiliations:** 1 Department of Botany and Plant Physiology, University of Life Sciences in Lublin, 15 Akademicka St., 20-950 Lublin, Poland; ewelina.chrzanowska@up.lublin.pl; 2 Department of Medicinal Plant and Mushroom Biotechnology, Faculty of Pharmacy, Jagiellonian University Medical College, 9 Medyczna Str., 30-688 Kraków, Poland; halina.ekiert@uj.edu.pl; 3 Faculty of Medicine, Institute of Medical Sciences, The John Paul II Catholic University of Lublin, 1H Konstantynów Str., 20-708 Lublin, Poland; lukasz.pietrzyk@wp.pl

**Keywords:** chemical composition, biological activities, γ-linolenic acid (GLA), essential oils, rosmarinic acid, anthocyanins, shikonin, silicon dioxide, cosmetic applications

## Abstract

One of the challenges of the pharmaceutical and cosmetic industries is to deliver biochemical compounds that can be advantageous for the skin. Research on Boraginaceae taxa has confirmed their use in traditional medicine and proved the potential biological importance of various molecules in cosmetology. The main classes of valuable compounds associated with Boraginaceae taxa are fatty acids, including γ-linolenic acid, essential oils, phenolic acids (e.g., rosmarinic acid), flavonoids, anthocyanins, tannins, and saponins. Highly specific are naphthoquinone pigments (including shikonin) and allantoin. Another distinguishing feature is the accumulation of silica (silicon dioxide) in trichomes. Some taxa produce mucilages. However, pyrrolizidine alkaloids (PAs) with toxic properties are also found (mainly in *Symphytum* spp.); therefore, their applications should be avoided. Extracts or individual compounds of Boraginaceae plants are characterized by antioxidant, anti-inflammatory, antiseptic, anti-irritant, antiaging, and photoprotective activities. Boraginaceae products are widespread in the cosmetic industry as ingredients of creams, balms, lotions, gels, shampoos, lipsticks, perfumes, and deodorants. The most valuable for the cosmetic industry are raw materials obtained from the genera *Alcanna Anchusa*, *Arnebia*, *Borago*, *Buglossoides*, *Cerinthe*, *Cordia*, *Echium*, *Ehretia*, *Eriodictyon*, *Glendora*, *Lappula*, *Lithospermum*, *Lycopsis*, *Macrotomia*, *Maharanga*, *Mertensia*, *Messerschmidia*, *Myosotis*, *Omphalodes*, *Onosma*, *Pulmonaria*, *Rindera*, *Symphytum*, *Trachystemon*, and *Trigonotis*. Further research should focus on the search for active substances in other plants of the family.

## 1. Introduction

In the 21st century, significant development of the cosmetic industry is noted, with a 23% increase in the number of cosmetic products on the overall consumer product market [1]. Consequently, there is a growing demand for high-quality products, including those based on plant raw materials [2,3].

In the last few years, many traditional medicinal plants have been analyzed using advanced methods (e.g., HPLC, MS, FTIR), leading to the discovery of various promising compounds for use in modern cosmetic formulations (e.g., [4,5,6,7,8]).

The Boraginaceae family (Euasterids I clade; APG IV system, 2019; the borage or forget-me-not family) is represented by approximately 148 genera and more than 2500 species (annuals, biennials, perennials, rarely trees, shrubs, or lianas) [9]. Stems and leaves of these plant species are covered by dense trichomes (glandular and non-glandular) [10]. The species are mainly native to Europe, Africa, and Asia (India, Iran). Most species are distributed worldwide; however; endemic species with the location restricted to, e.g., Sardinia, Anatolia, and Uzbekistan, are also known [11,12]. The family includes both rare species with threatened categories (e.g., *Echium russicum* CR in Poland or *Rindera umbellata* CR in Moldova) and species with the invasiveness status (e.g., *Anchusa officinalis*, *Echium vulgare*, and *E. plantagineum* in America) [13,14].

Boraginaceae taxa have more than 2000-year history of use (both internally and externally) in traditional medicine, mainly in the regions where they occur naturally and are commonly available [5]. Currently, their ethnopharmacological properties have been proved by chemical analyses, and the interest in these species has increased due to the content of biologically active molecules that can modulate the function and condition of the human organism and improve the appearance of the skin [2,3,8,15,16,17,18,19,20,21,22]. The therapeutic effect is mainly related to biologically active compounds, e.g., fatty acids, essential oils, phenolic acids, flavonoids, anthocyanins, tannins, naphthoquinones, saponins, allantoin, mucilages, pyrrolizidine alkaloids, and silica (silicon dioxide) isolated from Boraginaceae plant raw materials (e.g., [23,24,25]). Multiple clinical studies have demonstrated the advantageous effects of Boraginaceae extracts in relieving inflammation or alleviating symptoms in various disorders (gastrointestinal, rheumatoid arthritis, atopic dermatitis, eczema, psoriasis) [21,26,27,28]. Moreover, extracts have the ability to destroy, inhibit, or prevent the growth of microorganisms [29]. Numerous anticancer agents have also been listed [6,30]. However, compounds with toxic properties are also described. The toxic effect may be related to the presence of pyrrolizidine alkaloids (PAs) (e.g., [5,31]).

Given the numerous health potentials of Boraginaceae species and their common use worldwide, it is necessary to gather data available in the literature in order to identify areas of relevant research and main gaps in the knowledge and indicate central directions for future investigations. In this review, we tried to show the potentials of Boraginaceae species belonging to the genera *Alcanna Anchusa*, *Arnebia*, *Borago*, *Buglossoides*, *Cerinthe*, *Cordia*, *Echium*, *Ehretia*, *Eriodictyon*, *Glendora*, *Lappula*, *Lithospermum*, *Lycopsis*, *Macrotomia*, *Maharanga*, *Mertensia*, *Messerschmidia*, *Myosotis*, *Omphalodes*, *Onosma*, *Pulmonaria*, *Rindera*, *Symphytum*, *Trachystemon*, and *Trigonotis*, with special attention paid to their usefulness for the cosmetic industry. In particular, we focused on chemical molecules and their biological activities important for skin conditioning, care, and protection.

## 2. Methods

In this study, all the data were obtained from peer-reviewed articles available in the following databases: Google Scholar, ResearchGate, PubMed, Elsevier, Springer, ScienceDirect, and Wiley Online Library. We used the combination of the following keywords: “Boraginaceae”, “plant species names—*Symphytum officinale*, *Borago officinalis*, *Anchusa* spp., *Echium* spp., and others”, “names of chemical molecules”, “cosmetics”, and “herbal medicine” for searching in these databases. Chapters and books were also checked. The most representative references used were written in English. The following steps outlined by Arksey and O’Malley [32] were performed: (i) article scheme development and question formulation; (ii) recognition of relevant papers; (iii) selection and grouping of papers; (iv) data gathering, summarizing, organizing, and interpretation of available research; (v) manuscript drafting and final editing. The number of articles identified from the databases was 264. Due to duplication and repetitions, 46 articles were excluded from this list. Finally, 218 articles were used in this review.

Plant names follow The World Flora Online database [www.worldfloraonline.org/, accessed on 5 September 2024].

## 3. Boraginaceae Species in Ethnobotany

Plant use has been varying over time as a result of changes in the availability of different species in particular geographical regions. The practical use of plants in medicine or cosmetology is also influenced by the spread of knowledge and changes in crops grown by ethnic communities [33,34]. The members of Boraginaceae have a long-standing history of use for several arrays of purposes, including nutrition, beverages, flavorings, fragrances, and cosmetic and medicinal products [35,36]. In traditional medicine, they have been used in European, Asian (Iranian, Chinese, and Hindu), and American countries (Brazil, Mexico, USA) since ancient years [5,11,27,37]. Extracts, tinctures, and infusions are among the ways to transform raw materials into a form that can be used [38]. Various organs or parts of Boraginaceae plants (leaves, flowers, roots, fruits, bark, and wood) can be used as raw materials in therapies and cosmetics [39]. For example, *Symphytum* extracts have been applied for muscle pain mitigation, wound healing, and skin inflammation [40,41]. In turn, fresh herb juice of *Borago officinalis* has been commonly applied to treat respiratory problems, lung diseases, sore throat, arthritis, and skin disorders or to alleviate sadness [34,42]. In the Caribbean Islands, *Cordia alliodora* seeds in the form of powder or ointment have been used for skin diseases [36] and the references therein, and *Arnebia euchroma* ointment has been applied for burn wound healing in Asia [39].

### 3.1. Primary and Secondary Molecules in Boraginaceae

Plant-derived biologically active compounds are produced in a sequence of chemical reactions called a metabolic pathway, classified as (i) primary metabolism and (ii) secondary metabolism. Primary metabolism (=central metabolism) covers basic reactions and pathways that are absolutely necessary for the growth, development, and reproduction of cells. They maintain cellular homeostasis and the function of whole plant individuals [43]. Primary metabolites (sugars, amino acids, nucleotides, proteins, organic acids) are highly conserved and are generally found across different plant species [43,44,45]. Among Boraginaceae representatives, fatty acids are the most promising ingredients [12,46].

Secondary metabolism is involved in plant adaptation and mediation of the plant–environment interaction. Secondary metabolites (e.g., phenolics, terpenoids, and S- and N-containing molecules) are synthesized from primary metabolite precursors [47]. Compared to primary metabolism, the pathways of secondary metabolism are species-specific [48]. They have no direct role in the growth, development, and reproduction of plants but have multiple significant functions, e.g., defense against biotic stressors (herbivores, insects, and other pathogens) and enhancement of resistance against abiotic stressors (drought, UV radiation, salinity, low or high temperature). The most promising secondary metabolites in Boraginaceae taxa include essential oils, phenolic acids, flavonoids, anthocyanins, tannins, naphthoquinone pigments (shikonin and its derivatives), saponins, and allantoin [4,18,21,36,37,49,50,51,52,53] (Table 1 and Table 2). There is some controversy regarding whether certain chemical components belong to primary or secondary metabolism. As argued by some authors, mucilages belong to primary metabolites because of their sugar mixture composition. In contrast, other authors classify these compounds as secondary metabolites because their biosynthesis is species-specific [54]. In the current review, we present the information about mucilages in the section dedicated to secondary metabolites. Specific secondary phytochemicals are widely used in human food (flavorings, pigments), medicine (drugs, supplements), and cosmetology industry (volatiles, pigments) [47,55,56]. Individual chemical molecules (fatty acids, anthocyanins, naphthoginones, mucilages, tocopherols, and pyrrolizidine alkaloids) as well as their composition and concentration were found to have great taxonomic value in the Boraginaceae family [57,58,59,60,61].

### 3.2. Primary Metabolites

#### Fatty Acids

**Figure 1:**
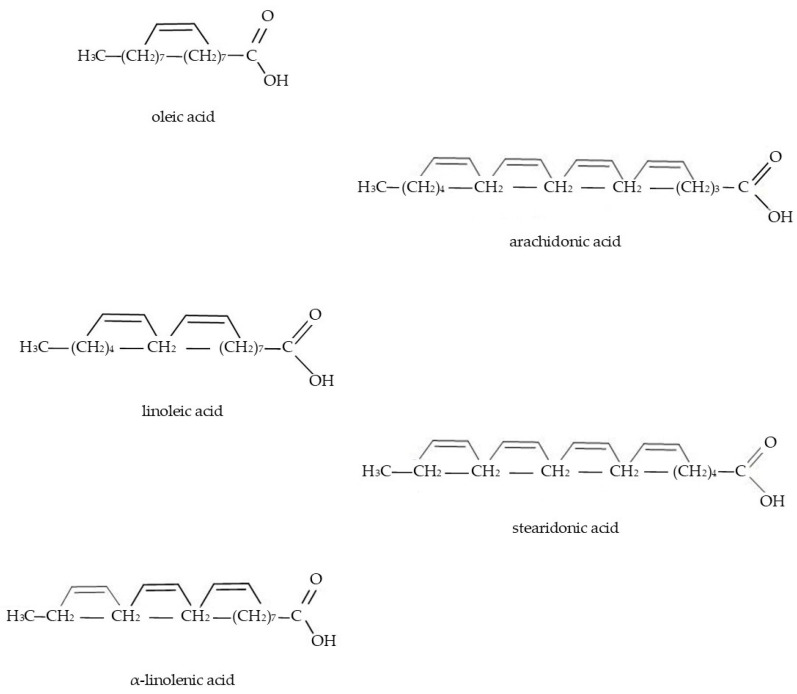
Chemical structures of fatty acids present in Boraginaceae plants.

In general, Boraginaceae taxa are well-known sources of fatty acids, which possess chemotaxonomic significance [54], Figure 1. For example, Özcan [12] screened 24 species from the Turkish flora and documented the total content of fatty acids ranging between 5 and 36% in their seeds. In Spain, a high amount of fatty acids, up to 21.1%, was detected in *E. asperrimum* seeds [80]. Considering fatty acid profiles, Boraginaceae taxa are rich in unsaturated fatty acids (Ω–6), e.g., arachidonic acid (AA, 20:4n–6; 15–17%) and linoleic (LA, 18:2n–6; 1.4–68.4%) [12,36,47]. Monounsaturated fatty acids, e.g., oleic acid (OA, 18:1n–9; 14–16.6%) and erucic acid (EA, 22:1n–9; 4–8.2%), are equally abundant [12]. Among polyunsaturated fatty acids, the highest amounts of α-linolenic acid (ALA; 18:3n–3; 12–43%), stearidonic acid (SA, 18:4n–3; 0.02–14.5%), and the unique γ-linolenic acid (GLA, 18:3n–6, 2–72%) were identified [12,37,56,80,99].

High content of GLA was found in *S. officinale* (16–72%) and *B. officinalis* (up to 25%) seeds [12,21,80,81]. GLA was also detected in the seeds of *Echium* spp., *Anchusa* spp., and *Trachystemon orientalis* [12] as well as in *Lithospermum latifolium*, *Pulmonaria officinalis*, and *E. plantagineum* [12,49,100,101]. Recently, *Mertensia* seeds have been indicated as sources of GLA-rich oil [80]. Other organs are a potential source of GLA as well. For example, *B. officinalis* contained approx. 2.5% of GLA in the leaves, and its concentration in the petals was approx. 16% [79].

It is well recognized that fatty acids, e.g., Borago oil (BO), play a significant role in maintaining the integrity of the stratum corneum. The target effects vary depending on the application form (internal/external), the dose used, and the duration of application [21]. Primarily, BO is effective in mitigating skin dryness [19]. For example, skin dryness was reduced by 14–40% in atopic dermatitis patients after two months of BO dietary supplementation (360 mg or 720 mg a day) [15]. This action is related to supporting the development of the skin barrier, which results in moisturizing the skin surface, increasing the water volume in the stratum corneum, and preventing water loss through the epidermis [TEWL] [15,49,102]. The hydration of the skin is presumably due to the participation of GLA in the increased synthesis of ceramides [16]. At the same time, GLA alleviates inflammation symptoms (e.g., eczema, skin redness, atopic dermatitis, acne), contributing to the comprehensive regulation of the immune system [16]. The advantageous clinical effect is likely associated with the inhibition of potent mediators of inflammation, including cytokines (Il-6 (interleukin-6) or TNF-α (tumor necrosis factor-alpha) [28,103]. It is well known that BO supports free radical scavenging; therefore, it has antiaging properties [104]. Positive effects of diets rich in BO on collagen synthesis have also been reported; consequently, reduction of skin wrinkles and improved skin texture was documented [19]. Moreover, BO significantly decreased skin melasma by inhibiting tyrosinase activity in melanocytes and contributing to the reduction of melanin synthesis [105]. Visual effects of whitening melasma spots were observed after 6–8 weeks of topical application of a cream containing 1% of BO. Therefore, BO is recommended for skin care and repair formulations and is an ingredient in cosmetics intended for dry and sensitive skin care, skin with acne, seborrheic dermatitis, and atopic dermatitis [2,50,101,106].

## 4. Secondary Metabolites

### 4.1. Essential Oils

**Figure 2:**
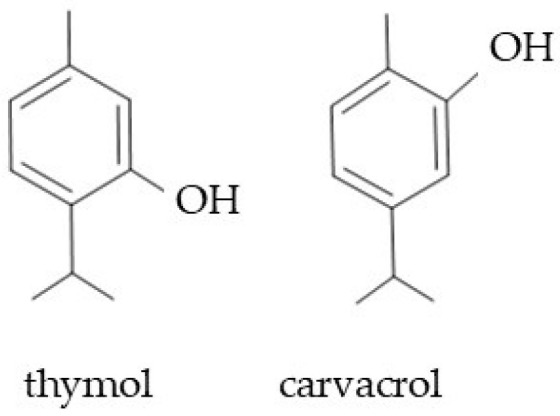
Chemical structures of simple phenolic compounds in essential oils of *Echium amoenum*.

Essential oils (EOs) are mixtures of aromatic volatile substances of various chemical classes and properties [107]. In Boraginaceae representatives, EOs are mainly found in glandular trichomes located on the surface of flowers, leaves, fruits, and seeds [4,108]. Essential oils differ from each other in their chemical composition, although there are some chemical groups responsible for their properties, e.g., simple phenolic compounds and different subgroups of terpenes (e.g., [53,70,72]). The qualitative and quantitative characteristics of essential oils are species-dependent [71,72]. In the same species, the essential oil composition and the concentration of their compounds may vary depending on the geographical location, plant age, and cultivation (soil moisture, soil trophy) and weather conditions (light, humidity, temperature) [109,110]. Most Boraginaceae EOs have a very complex chemical composition, ranging from several to over a hundred isolated chemical molecules [37,70,71,72,111,112]. The most frequently identified ingredients responsible for the biological properties of Boraginaceae EOs are simple phenolic compounds (thymol, carvacrol) (Figure 2), monoterpenes (α-pinene, eucalyptol, α- and-β-phellandrenes), diterpenes (phytol), sesquiterpenes (α-bisabolol, α-humulene, trans-caryophyllene, alloaromadendrene, α-eudesmol, δ-cadinene, β-caryophyllene, β-gurjunene, β-ionene), alkanes (heptane, hentriacontane, eicosane), esters (di-isobutyl phthalate, methyl salicylate), benzopyrones (coumarins), and aldehydes (nonanal, benzeneacetaldehyde = hyacinthin) [70,72,73,109,111,112,113,114].

In particular, a high concentration of biologically active molecules was identified in EOs from *Auxemma glazioviana* (94%) [71], *Myosotis arvensis* and *M. palustris* (42.7–45.8%) [70], *Anchusa italica* (32.6%) [74], *Echium amoenum* Mey. (7.5–19.5%) [72], and *Varronia curassavica* (syn. *Cordia verbenacea*) (10–15%) [109]. Aromatic volatile compounds are also characteristic for *Symphytum kurdicum* and *S. asperum* [75], other *Cordia* species [36,76,84], and references cited therein, *Glendora rosmarinifolia* [113], and *Paracaryum bingoelianum*, a new species recognized in the flora of Turkey [73].

The EOs in the Boraginaceae species exert strong antioxidant, anti-inflammatory, and antiseptic (=antibacterial, antiviral, and antifungal) effects on the skin [2,62,71,77,78]. Molecules present in EOs have the ability to scavenge free radicals and prevent skin cell damage induced by reactive oxygen species (ROS) [115]. A very promising anti-inflammatory effect of Boraginaceae EOs is related to the documented reduction in the number of mediators responsible for the inflammation process (IL-6, TNF-α, COX-2) and regulation of the NF-κB pathway, which may reduce the effects of pathological skin inflammation [27]. For example, anti-inflammatory properties have been documented for α-bisabolol, which helps to alleviate inflammation symptoms, such as itching, pain, and swelling [116,117,118]. The α-bisabolol molecule is potentially non-allergenic and anti-irritant and is thus regarded as a safe ingredient for baby products [119]. Coumarins and β-humulene molecules are known to inhibit the action of tyrosinase [120]. Tyrosinase is an enzyme involved in melanogenesis. It is responsible for the formation of skin pigment [121]. Inhibition of the action of tyrosinase may be effective in the fight against emerging skin discolorations [122]. When applied topically to the skin, coumarins show photo-protective actions [123] and exhibit great potential for use in whitening cosmetics [124]. Recent results suggest that EOs isolated from Boraginaceae plants (e.g., *Paracaryum bingoelianum*, *Cordia* spp.) contain bioactive compounds with antitumoral activity (e.g., β-caryophyllene, α-humulene, α-pinene) [36,73].

The antimicrobial activity has been proven in in vitro tests; for example, the β-phellandrene molecule isolated from *Cordia* species and *Paracaryum bingoelianum* inhibited the development of *Bacillus* sp., *Staphylococcus aureus*, and *Escherichia coli* [125]. The α-pinene molecule revealed an inhibitory effect on the growth of fungi, e.g., *Rhizoctonia solani* and *Colletotrichum lindemuthianum* [126].

It has been evidenced that Boraginaceae EOs are crucial for delaying the skin-aging process and are therefore suggested as ingredients in antiaging cosmetics intended for mature skin [71,127]. In the cosmetics industry, essential oils add a pleasant scent to cosmetic products, e.g., perfumes, deodorants, creams, balms, lotions, lipsticks, and hair care cosmetics (shampoos, gels) [2,128,129].

### 4.2. Phenolic Compounds

#### 4.2.1. Phenolic Acids

**Figure 3:**
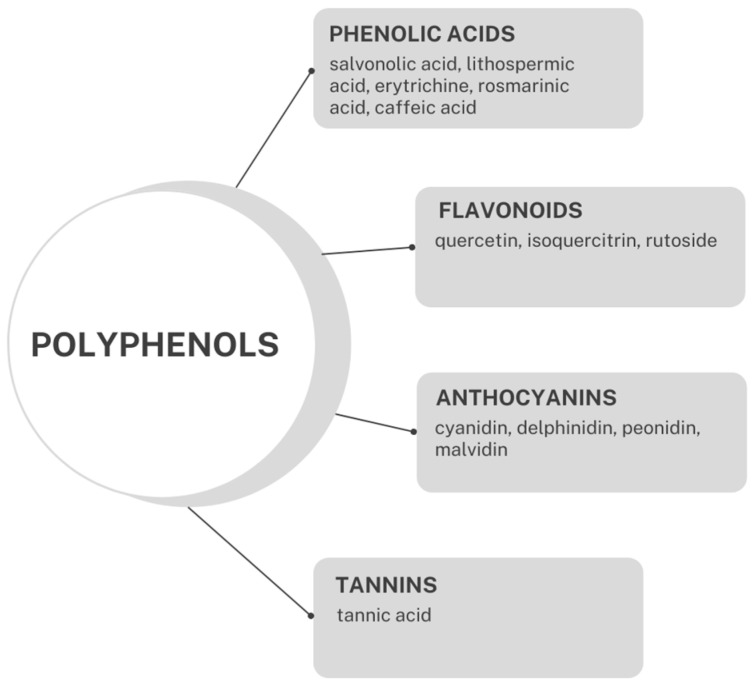
Main phenolic subgroups found in Boraginaceae.

**Figure 4:**
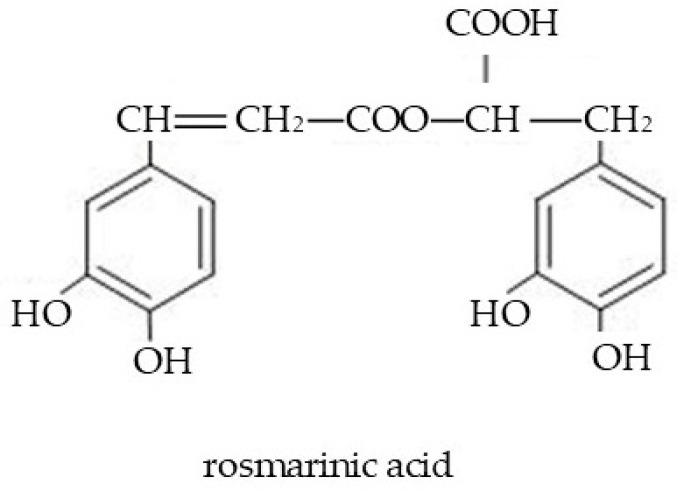
Chemical structure of rosmarinic acid commonly found in many species of Boraginaceae.

Phenolic acids are another group of compounds found in extracts obtained from Boraginaceae members. For example, *Anchusa officinalis*, *A. italica*, *Echium vulgare*, and *E. russicum* contain diverse phenolic molecules (e.g., salvonolic acid, lithospermic acid) (Figure 3) [115,130,131,132]. The content of total phenolic acids in *A. officinalis* extracts has been reported to range from 6.60 to 116.42 mg GAE/g of dry extract [131]. Among phenolic acids, p-hydroxybenzoic acid, hydrocaffeic acid, and chlorogenic acid have also been found in roots or shoots of Boraginaceae species [4]. However, the most common constituent of Boraginaceae taxa is rosmarinic acid (RosA; Figure 4) (=ester of caffeic acid and α-hydroxy-dihydrocaffeic acid; 3.4-dihydroxy-phenyllactic acid) [133,134]. In particular, high amounts of RosA (2.5–3.3% dry wt) have been documented in shoots of *A. azurea*, *A. undulata*, *Pulmonaria mollis*, and *Buglossoides purpurocaerulea*. However, other species, namely *Cerinthe minor*, *Omphalodes verna*, *P. obscura*, *Symphytum cordatum*, *Mertensia maritima*, *Ehretia obtusifolia*, *Rindera graeca*, and *Trachystemon orientalis*, contain RosA as well [4,90,91,92,93,135]. In the *Rindera* genus, caffeic acid and its derivatives, rutin, and quercetin-3-rutinoside-7-rhamnoside (an unusual triglycoside) were identified [135]. Among flavones, apigenin and luteolin were isolated for the first time in Boraginaceae plants by Petreska Stanoeva et al. [132].

RosA is classified as a strong antioxidant and anti-inflammatory molecule [115,131,132]. It has been proved that the antioxidant activity of phenolics depends on solvent polarity [136]. In the skin, RosA can block or neutralize free radicals [94]. The mechanism of the antioxidant effect may be related to inhibition of the MAPK//NF-κB pathway, which downregulates the expression and activity of nitric oxide synthase [137]. Due to its antioxidative properties, RosA is considered effective in the care of wounded skin [138]. Wound healing is a complex process that aims to rebuild the damaged skin barrier [139]. There are reports indicating that the use of rosemary cream supports faster shrinkage of wounds and accelerates their healing [140]. As reported by Lee et al. [23], RosA reduces chronic inflammation common in atopic dermatitis. The anti-skin cancer effects of RosA have also been described reviewed in [116,118]. The potential antimelanoma effect of RosA is related to its involvement in the induction of the melanogenesis process and its contribution to the increase in the melanin concentration [141,142,143]. It is suggested that RosA regulates the signaling pathway (PKA/CREB/MITF) of the development of melanocytes in the skin [143,144]. RosA attenuates skin tissue damage caused by reactive oxygen species resulting from exposure to UV rays and responsible for the induction of uncontrolled cell division in the process of carcinogenesis [23,134,144,145]. Other possible pathways for the involvement of RosA in reducing skin cancer development are related to its ability to inhibit the overexpression of cyclooxygenase-2 (COX-2) and other pro-inflammatory products (e.g., prostaglandin E2; PGE2), known as critical mediators of inflammatory response [146,147]. The beneficial role of RosA in the inhibition of uncontrolled growth of skin cancer cells was evidenced by Osakabe et al. [148]. However, the therapeutic effect of RosA may be impaired due to its poor bioavailability [149]. In order to increase the stability of RosA in cosmetic preparations, microencapsulation technology is implemented, which ensures the use of the RosA potential [150]. An important biological feature of RosA is protection against excessive transepidermal water loss and acceleration of skin hydration; therefore, the molecule is used in antiaging skin treatments [151].

#### 4.2.2. Flavonoids

**Figure 5:**
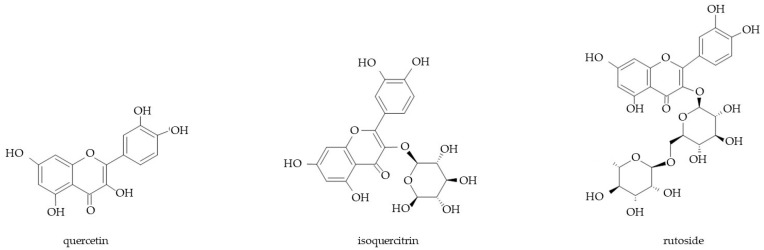
Chemical structures of flavonoids found in various organs of Boraginaceae.

Flavonoids, i.e., polyphenols with a variable phenolic structure, have also been isolated from various organs of Boraginaceae taxa (Figure 5). Quercetin glycosides (rutoside and isoquercitrin) have been reported in *Anchusa azurea* var. *azurea* herb [152] and *Lithospermum officinale* leaves [153].

**Figure 6:**
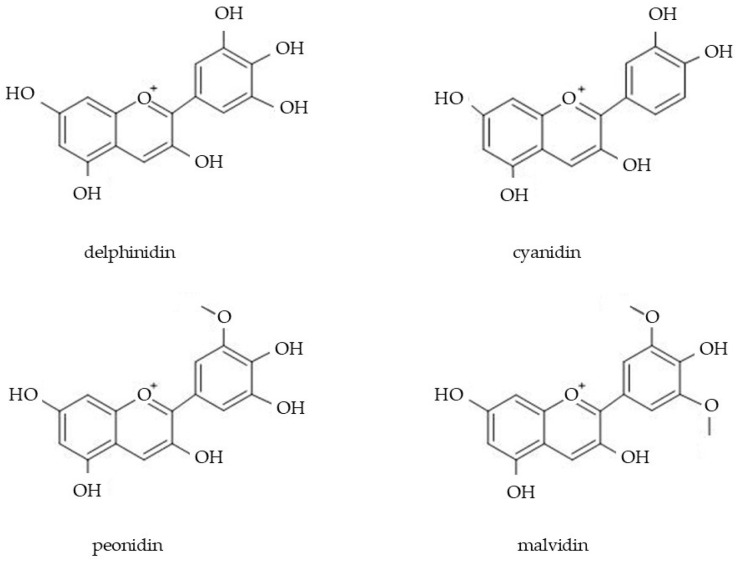
Chemical structures of anthocyanins present in *E. plantagineum* pollen.

Flavonoid molecules are primarily used in cosmetics due to their antioxidant, ant-inflammatory, and soothing properties [154]. The molecules also add a pleasant color to hair, face, and body cosmetic products and cleaning products [155]. Moreover, rutin exhibits a protective effect on blood capillary vessel walls, improves blood circulation, and prevents platelet aggregation [156]. Quercetin has the ability to protect melanocytes and keratinocytes from oxidative stress and has shown positive effects in the treatment of pigmentary skin disorders [157]. Flavonoids of Boraginaceae taxa are also indicated as valuable photoprotective molecules for use in modern cosmetic formulations as they prevent the absorption of ultraviolet A and ultraviolet B radiation [158,159]. Moreover, there are suggestions of the positive effect of flavonoids in the treatment of psoriasis [157]. Anthocyanins are water-soluble phenolic pigments accumulated in vacuoles, very common among plants [160,161]. Anthocyanins are classified by some authors as a subclass of flavonoids and are characterized by color variability (from red through purple to blue) related to changes in cell sap pH [162]. These molecules are synthesized in the phenylpropanoid pathway; i.e., they are derivatives of phenylalanine and tyrosine [163]. Anthocyanin molecules have a flavylium cation (AH+) that acts as an acid [164]. Their stability is also affected by light and temperature [162]. Currently, more than 630 anthocyanins have been identified [165]. In Boraginaceae species, high concentrations of common anthocyanins—cyanidin and delphinidin 3-glucosides—were detected in *Anchusa arvensis* and *Nonea caspica* [62]. A dark-red or purple anthocyanin petunidin-3-O-rutinoside as well as delphinidin, cyanidin, peonidin, and malvidin were found in the pollen of *Echium plantagineum* (Figure 6; in total 40–80 mg/100 g of anthocyanins) [63,64]. In general, bee pollen exhibits great potential for use in cosmetology as it contains numerous active ingredients (e.g., flavonoids). However, the properties of pollen are highly variable because they depend on many factors (e.g., botanical origin, harvesting and storage conditions) [166]. For example, “Spanish bee pollen” is mainly collected from *E. plantagineum*; however, pollen from other species is also present in Iberian pollen loads (e.g., *Quercus* sp., *Cistus* sp.) and differs in its biological activity [63].

In *E. amoenum* grown in Iran, the anthocyanin content was 104 mg/100 g [65]. The potential use of anthocyanins in cosmetics is related to their high bioactivity and lack of toxicity [66]. Therefore, extracts of anthocyanins are considered to be safe cosmetic ingredients [167].

With the small size of their molecules, anthocyanins can easily penetrate the skin and are emerging as one of the most promising ingredients in cosmetology [67]. The advantageous biological features of anthocyanins important for skin care and protection include antioxidant [68], anti-inflammatory [163], and bacteriostatic properties [168]. An important feature of anthocyanins is the ability to absorb or block UV energy; therefore, they are widely used in sunscreen products to minimize solar-related skin damage [69].

The protective mechanism of anthocyanins is associated with the well-established prevention of UV radiation penetration, alleviation of oxidative stress and inflammation, enhancement of DNA repair, inhibition of the extracellular matrix degradation in the dermis, and inhibition of skin elasticity loss [162]. Anthocyanins have been shown to control the composition of the gut microbiota and may play a central role in the prevention of inflammation-mediated skin diseases (e.g., atopic dermatitis, rosacea, and psoriasis) [161]. Therefore, extensive research is suggested on the effect of anthocyanins on the bidirectional relationship between the gut microbiome and skin health (i.e., gut–skin axis effect) [169]. Anthocyanins are also of great interest due to their potential use as colorants in cosmetics; however, their application is still difficult due to their low ability to dissolve in oils and susceptibility to pH changes [170]. Therefore, future research on stabilization of these pigments in cosmetics is suggested [66].

#### 4.2.3. Tannins

Tannins are also classified among polyphenolic biomolecules [171]. The chemical structure of the molecules (the presence of hydroxyl groups) enables them to establish a permanent link with proteins and carbohydrates [169]. In plants, tannins protect against external biological and environmental threats, e.g., against microbial and herbivore attacks or against stress caused by drought/salinity. Tannins are found in various parts of plants (roots, stems, bark, fruits, leaves, and seeds). In the Boraginaceae family, the genera *Anchusa*, *Echium*, *Pulmonaria*, and *Symphytum* are characterized by the presence of tannins [75,97]. It has to be stressed that the tannin content is species-specific and may vary significantly within a species depending on environmental conditions [36]. For example, a high amount of tannins (38 mg/g of dry matter) was found in *Echium amoenum*, whereas a lower value (26 mg/g of dry matter) was documented in *E. russicum* [36]. In *Pulmonaria mollis*, the tannin content amounted to 7.1 mg/g of dry matter of the raw material [97].

**Figure 7:**
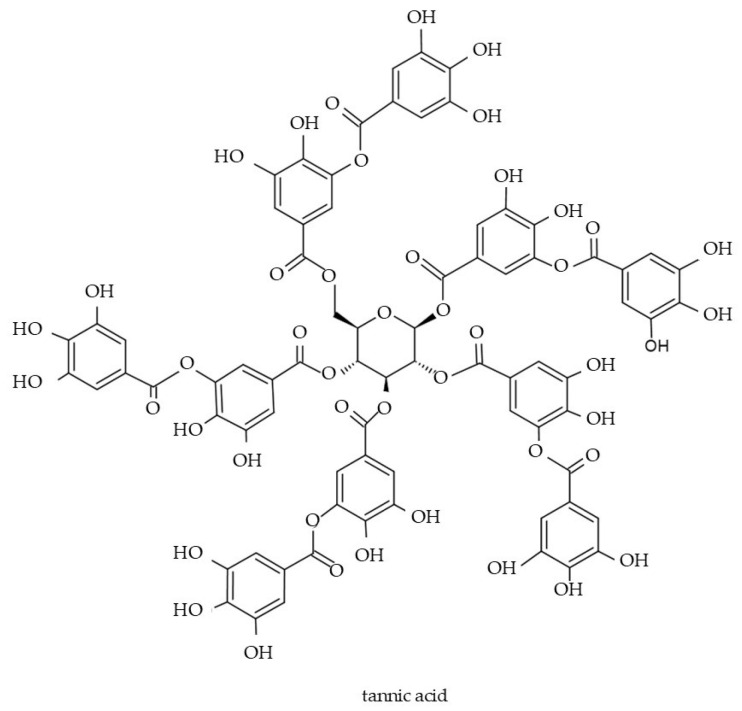
Chemical structure of tannic acid.

A characteristic property of tannins is their astringent function [172]. Moreover, tannins exhibit other important biological properties, i.e., ROS reduction and antimicrobial activity [173]. The activity of tannins against ROS is related to the protection of cell membranes from peroxidation of lipid molecules and the prevention of DNA structure damage [172]. Tannins effectively prevent premature skin aging and protect the skin against cancer development [169]. Their high antibacterial activity makes them potentially desirable in cosmetics intended for acne-prone skin as they may help reduce acne breakouts and blemishes [169]. Tannic acid (TA) (Figure 7), a form of hydrolysable tannins, is especially valuable in cosmetic industry [174]. It has been proved that the molecule is effective in skin protection against ultraviolet radiation and reduces the features of photoaging [98]. Moreover, TA supports the treatment of atopic dermatitis, reduces keratinization of the epidermis, accelerates wound healing, and soothes pathological lesions [98]. Currently, efforts are focused on improving the antioxidant properties of tannin molecules for cost-effective and eco-friendly application in the cosmetics industry, including the replacement of synthetic preservatives [174].

### 4.3. Other Subgroups of Secondary Metabolites

#### 4.3.1. Naphtoquinone Pigments

**Figure 8:**
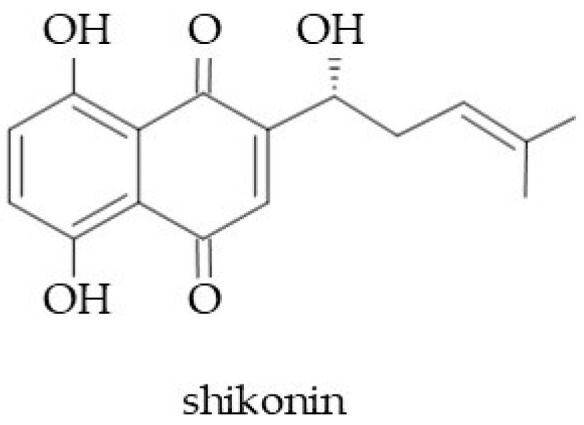
Chemical structure of shikonin.

Naphthoquinones are a subclass of chemical compounds classified among quinonoids (quinones). The spatial arrangement of their chemical structure is characterized by the addition of another benzene ring connected to para-benzoquinones [8]. The presence of quinones (pigments) is a characteristic attribute of several Boraginaceae taxa. Of these dyes, shikonin (red pigment; Figure 8) was the first to be isolated from the roots of the Chinese plant *Lithospermum erythrorhizon* in the second decade of the 20th century [175]. To date, shikonin and its derivatives have been extracted from *Borago* spp., *Alcanna* spp., *Arnebia guttata* Bunge, *Arnebia euchroma*, *Maharanga* spp., *Lithospermum erythrorhizon*, *Onosma paniculatum*, *Onosma hookeri*, *E. vulgare*, *E. russicum*, and *E. italicum* [4,6,7,8,96,176]. Recently, shikonin-type naphthoquinones have been reported in *Rindera graeca*, an endemic Greek plant [25].

It has been proven that shikonin and its derivatives have multidirectional biological properties, e.g., antioxidant, anti-inflammatory, antiallergic, antibacterial, antiviral, antifungal, and antithrombotic activity [6,7,30,96,177,178]. These properties make the molecules very promising compounds for wide use in pharmaceutical and cosmetic industries [25]. The molecule can accelerate the healing of wounds and burns; therefore, it is particularly helpful in plastic surgery and esthetic cosmetology [7]. Moreover, it improves the functioning of the skin barrier, expands skin hydration, regulates skin immunology, reduces and prevents skin inflammation, and supports the development of keratinocytes [24,30]. Shikonin and its derivatives also effectively reduce the bothersome symptoms of atopic dermatitis [179] and support the treatment of psoriasis [180].

The mechanisms of antioxidant action result from the presence of a phenoxy group in shikonin, thanks to which reactive oxygen species are neutralized [24]. A trend that has been developing very rapidly recently is the study of the mechanisms of the anticancer activity of shikonin and its derivatives (e.g., acetylshikonin) [181]. These compounds showed cytotoxic action against various cancer cells (lung, colon, prostate, breast, skin) [175,182]. Possibly, the inhibition of skin cancer growth is related to the induction of cancer cell apoptosis via the MAPK pathway [183].

#### 4.3.2. Saponins

The other constituents in Boraginaceae are saponins—natural chemicals comprising an aglycone unit (triterpene or steroid) linked to carbohydrate chains (hexose and/or uronic acid) [11,184]. The INCI list contains several hundred registered patents showing the possibility of using saponins in cosmetics [185]. Among Boraginaceae taxa, oleanane-type saponins (e.g., acetylanchusoside-9, malonylanchusoside-2, malonylanchusoside-7) were isolated from *A. officinalis* L. roots [95]. In turn, triterpenoid saponins leontoside-A and B and symphytoxide-A were extracted from *S. officinale.* Similarly, the presence of various saponins was found in *E. italicum* L. stems [11] and *Trichodesma indicum* Linn. roots [186]. However, saponins detected in *Cordia piauhiensis* turned out to be inactive [184].

It is known that saponins enhance blood flow in skin capillaries and are employed in treatments reducing cellulite symptoms [27]. These biomolecules also show antibacterial activity [187]. In the cosmetology industry, saponins are regarded as natural alternatives to surfactants or emulsifiers [188]. Traditionally, these molecules are used as natural additives in shampoos and shower gels as well as moisturizing ingredients in creams [2,188].

#### 4.3.3. Allantoin

**Figure 9:**
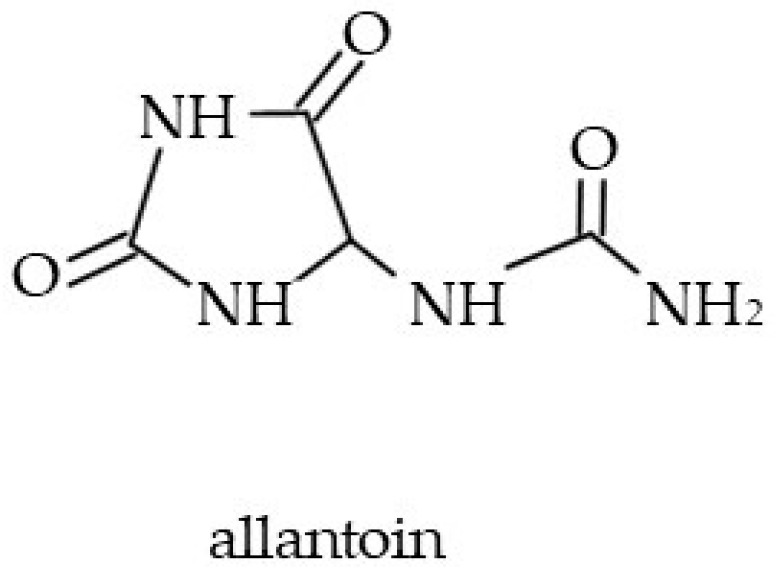
Chemical structure of allantoin.

Allantoin (2,5-dioxo-4-imidazolidinyl)urea; Figure 9 is a chemical compound produced from uric acid [189]. A high concentration of allantoin was detected in *Symphytum officinale* and *S. cordatum* [20,59,190]. *Echium italicum*, *E. russicum*, *Lithospermum anchusoides*, *Symphytum cordatum*, *Pulmonaria obscura*, and *P. mollis* (c.a. 2–3.5% dry wt) are other commonly known sources of allantoin. Lower amounts were found in *E. vulgare*, *L. officinale*, *Cynoglossum creticum*, *Omphalodes verna*, *Buglossoides purpurocaerulea*, and *Cerinthe minor* L. [4]. Allantoin was also identified in *Mertensia maritima* (3.7% dry wt) [50]. In folk medicine, it was used to encourage wound healing and various skin disorders [2]. Currently, allantoin is used in body and face care products at a concentration range of 0.5% to 2% [189,190]. It is regarded as a safe and hypoallergenic compound with skin smoothing and softening properties [159]. Presumably, allantoin acts via a reduction in the level of interleukins (e.g., IgE, IL-5); it therefore helps to reduce skin inflammation symptoms (redness, swelling, itching) [191]. At the same time, allantoin stimulates the proliferation of fibroblasts and contributes to the wound-healing process [60]. The effectiveness of this molecule for skin conditioning was reported for more than 1300 products [189].

#### 4.3.4. Mucilages

Mucilages belong to the group of carbohydrates and have a complex chemical structure [83]. Plant mucilages are a mixture of sugar molecules (e.g., rhamnose, galactose) along with other organic and inorganic compounds (vitamin C, lecithin). In terrestrial plants, they help to maintain and store moisture in tissues [84]. Among Boraginaceae plants, mucilages were described in *B. officinalis* [85], *E. amoenum* [86], *S. officinale* [87], and *C. dichotoma* [84]. These biomolecules possess many characteristics (stabilizing potential, viscosity enhancement, emulsifying effects, adhesive properties, extensive adaptability) which make them highly desirable by the textile, food, and cosmetic industry [192]. In cosmetics, mucilage biomolecules enhance the moisturizing, softening, and elasticizing effect on the skin [83]. Therefore, mucilages are used to combat skin diseases; they are suitable for the care of dry, dehydrated, sagging skin and skin with eczema [84]. Mucilages widen skin pores and are applied before cosmetic and medicine delivery to the dermis [88]. Due to the presence of phenolic compounds, mucilages also have antioxidant properties [89]. Additionally, they play an important role in the wound-healing process and limit wound infection by the creation of hydrogels on the basis of mucus and acting against microbes [84]. Topical application is proven to be most effective in the case of skin lesions, wounds, abscesses, varicose veins, rashes, and warts [193,194].

#### 4.3.5. Pyrrolizidine Alkaloids

Several studies have reported pyrrolizidine alkaloids (PAs) with strong toxic effects isolated from Boraginaceae species [195]. The best recognized are the alkaloids from different species of the genus *Symphytum* (*S. asperum*, *S. caucasicum*, *S. cordatum S. officinale*, *S. tuberosum*, and *S*. × *uplandicum*) [61]. Among pyrrolizidine alkaloids, lasiocarpine, lycopsamine, 7-acetyllycopsamine, asperumine, echimidine, intermedine, symlandine, and symphytine, including their related N-oxides, were detected with the highest quantities [5,20,31,51,196,197]. The total content of pyrrolizidine alkaloids in *S. officinale* L. depended on the plant organ. For example, it ranged widely from ca. 1300 to 8300 μg/g in roots and from 10 to 60 μg/g in leaves [198]. The alkaloid lycopsamine was isolated from *S. uplandicum*, whereas anadoline and echimidine were detected in *S. tuberosum* L. [199,200]. Another alkaloid, e.g., indicine-N-oxide (a derivative of L-ornithine), was detected in *Cynoglossum creticum* and *Heliotropium indicum*, whereas europine and ilamine and their N-oxides were found in *H. crassifolium* [201]. Diverse PAs were identified in *Echium sabulicola* ssp. *decipiens* and *Solenanthus lanatus* [55]. PAs are regarded as the most toxic among plant alkaloids [198,202]. They can trigger or promote carcinogenesis [203]. However, no adverse effects have been documented after external application, which suggests that the skin absorption of these compounds is negligible [40]. However, due to the possible toxic properties, the internal use of *Symphytum* extracts in therapy is restricted in many countries, e.g., in Poland, Germany, Denmark, Austria, Canada, and the USA [5]. The EU commission recommends external use for no longer than 4–6 weeks reviewed in [193]. Pyrrolizidine compounds do not appear on the INCI list of cosmetic compounds, but extracts made from plants that produce these compounds are used in cosmetic products [185]. Therefore, the possibility of the potential use of cosmetic products that may contain pyrrolizidine alkaloids should be based on a risk assessment and determination of a margin of safety indicating the maximum dose of a substance that the skin can be exposed to per day, considering that dermal administration is associated with 100% skin penetration by PAs [52,196].

## 5. Bioelements

### Silicon (Si) and Silicon Dioxide (SiO_2_)

**Figure 10:**
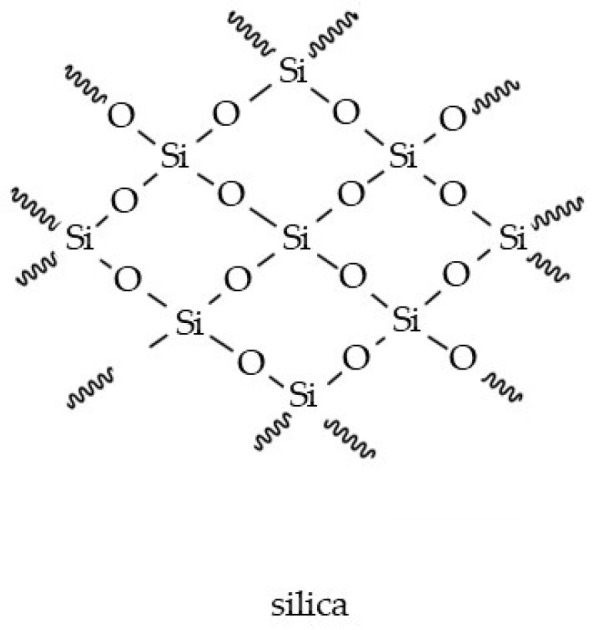
Chemical structure of silica (silicon dioxide).

Silicon is a microelement taken up by roots and accumulated in the form of SiO_2_ (silica, silicon dioxide, silicophytoliths; Figure 10) on the surfaces of cell walls and intercellular spaces [204]. Silicophytoliths are commonly found in trichomes on the surface of leaves/stems in Boraginaceae plants [10]. Mineralized trichomes with high concentrations of silicon are characteristic of *B. officinalis*, *E. vulgare*, and *S. officinale* [205]. In plants, silicon molecules are not necessary; however, they are beneficial for plant resistance to environmental conditions [204]. In humans, they are essential for proper bone mineralization and are involved in the growth of hair and nails [206]. Silicon is necessary for the synthesis of elastin and collagen and is thus responsible for the durability and flexibility of skin tissues [89]. Traditionally, silicon is used as a food supplement to strengthen nails, counteract hair loss, combat fungal infections, and treat acne (acne vulgaris, rosacea) [207]. Silicon also reduces capillary permeability and improves skin-wound-healing outcomes [88]. The possibility of synergistic action of silicon molecules with other molecules present in plants (e.g., flavones, tannins) means that silicon of plant origin exerts a better effect on the body than silicon of synthetic origin [207]. In cosmetics, the silicon molecule occurs mainly in the form of inorganic silicates (silicon and oxygen anions) and organic silanols (hydroxyl-containing compounds) [89]. They are used in cleansing cosmetics, toothpastes, and body scrubs [208]. Diverse forms of silicon can be applied to improve skin moisture by the enhancement of water binding in the skin; therefore, they are used in skin moisturizing and antiaging cosmetics [206]. Their water-resistant properties are desirable in sunscreens to ensure the adherence and formation of an appropriate amount of film on the skin surface [88].

## 6. In Vitro Production of Secondary Metabolites

The growing industrial demand for biologically active compounds has resulted in the development of methods of their production using modern biotechnology and genetic engineering [135,177]. Biotechnological production of desirable metabolites is of key importance because natural methods of obtaining appropriate amounts of raw material and its metabolites are problematic, e.g., due to the long wait time for a noticeable production capacity for plants [182,209].

Among others, plant cell culture technologies are widely involved to provide effective tools for delivering plant-derived molecules for industrial applications (i.e., food products, cosmetics, drugs) [210,211]. Examples of effective methods of increasing the bioactive phytochemical content in plant cells include the optimization of culture systems, elicitation, and genetic transformation [7,24].

Novel procedures for efficient commercial-scale, cost-effective technology for obtaining natural metabolites were introduced by Mibelle Biochemistry company, which developed effective methods of establishing callus cultures derived from *Symphytum officinale* roots [209]. The PhytoCellTec™ Symphytum (Mibelle Biochemistry, Buchs, Switzerland) product has the power to enhance the regeneration of epidermal cells and improve the function of the skin barrier [209].

Another cosmetic ingredient, shikonin, is commercially obtained by Mitsui Petrochemical Industries Ltd. (Tokyo, Japan) [209]. The shikonin production is performed in *Lithospermum erythrorhizon* cell cultures. The cell cultivation protocol covers two phases: (i) the suspension of cell cultures in the growth medium and (ii) the growth of cell cultures in the production medium using a bioreactor with an air stirrer to provide oxygen [177,212]. The in vitro production of shikonin using a two-stage culture system was also established for the manufacture thereof in cell cultures from *L. canescens* [213], *Echium plantaginatum* [214], *E. italicum* [215], *Arnebia euchroma* [216], and *Onosma paniculatum* [217]; however, the systems have not been commercialized yet.

Recently, research has been carried out to develop an effective technology for intensification of the biosynthesis of biologically active compounds in a culture of transgenic roots of *Rindera graeca*, a potential source of naphthoquinones [218].

## 7. Conclusions

This multidirectional study indicated the popularity of biologically active molecules contained in various parts of species belonging to the Boraginaceae family as cosmetic ingredients. The most valuable activities for skin care, protection, and conditioning are their antioxidant, anti-inflammatory, antibacterial, antiviral, antifungal, anti-irritant, antiaging, photoprotective, moisturizing, softening, and elasticizing properties. Detailed research of the CosIng database showed that 39 raw materials originating from 19 species have been accepted for use in cosmetics. Future research should focus on the identification of bioactive compounds in other Boraginaceae species, including rare and endangered species. Furthermore, since a multi-component mixture of bioactive compounds is present in plant material, optimization of extraction, isolation, and separation methodologies (using a combination of chromatographic and non-chromatographic techniques) is required. Since pyrrolizidine alkaloids (PAs) are common in Boraginaceae, the safe application of Boraginaceae derivative compounds in cosmetics requires the development of strategies for purification thereof. Considerable attention to cell cultures should also be paid.

## Figures and Tables

**Table 1:** Biologically active compounds found in the species of the Boraginaceae family and their effects on the skin.

Chemical	Species/Genera	Effect on the Skin	References
Allantoin	*Buglossoides purpurocaerulea*, *Cerinthe minor*, *Cynoglossum creticum*, *Echium italicum*, *E. russicum*, *E. vulgare*, *Lithospermum latifolium*, *L. officinale*, *Lindelofia anchusoides*, *Martensia maritima*, *Omphalodes verna*, *Pulmonaria mollis*, *P. obscura*, *Symphytum cordatum*, *S. officinale*	increase skin softness, strengthen the skin, accelerate wound healing	[4,20,51,60,61]
Anthocyanins	*Anchusa arvensis*, *Echium plantagineum*, *E. amoenum*, *Nonea capsica*	antioxidant effect, protection against UV radiation	[62,63,64,65,66,67,68,69]
Essential oils	*Auxemma glazioviana*, *Anchusa italica*, *Cordia species*, *Echium amoenum*, *Myosotis arvensis*, *M. palustris*, *Paracaryum bingoelianum*, *Symphytum asperum*, *S. kurdicum*,	influence the scent of the cosmetic, may act as a preservative, have anti-acne, antibacterial, and antioxidant properties	[2,62,70,71,72,73,74,75,76,77,78]
GLA	*Anchusa* spp., *Borago officinalis*, *Echium* spp., *Lithospermum latifolium*, *Mertensia* spp., *Pulmonaria officinalis*, *Symphytum officinale*, *Trachystemon orientalis*	improve hydrolipid barrier of skin, support the treatment of eczema, psoriasis, and acne	[12,15,21,49,79,80,81,82]
Mucilages	*Borago officinalis*, *Cordia dichotoma*, *Echium amoenum*, *Symphytum officinale* L.	soften and elasticize the skin, have a moisturizing effect, dilate pores before cosmetic treatments, have antimicrobial properties	[83,84,85,86,87,88,89]
Rosmarinic acid	*Anchusa azurea*, *A. undulata*, *Borago officinalis*, *Buglossoides purpurocaerulea*, *Cerinthe major*, *Echium italicum*, *Ehretia obtusifolia*, *Heliotropium amplexicaule*, *Lindelofia longiflora*, *Lithospermum* sp., *Mertensia maritima*, *Nonnea lutea*, *Pulmonaria mollis*, *Symphytum* sp., *Trachystemon orientalis*	antioxidant properties, support the fight against free radicals	[4,90,91,92,93,94]
Saponins	*Althaea officinalis*, *Echium italicum*, *Symphytum officinale*	foaming agents in body wash products and shampoos	[2,11,95]
Shikonin (red pigment)	*Arnebia euchroma*, *A. guttata*, *Borago* spp., *Echium italicum*, *E. russicum*, *E. vulgare*, *Lithospermum erythrorhizon*, *Onosma hookeri*, *O. paniculatum*,	reduce free radicals, moisturize, strengthen the skin barrier, important in the production of red lipsticks	[4,24,96]
Tannins	*Anchusa* L., *Pulmonaria* L., *Symphytum* L.	antioxidant and antiaging effects, alleviate symptoms of atopic dermatitis, support wound healing	[75,97,98]

**Table 2:** The list of ingredients derived from Boraginaceae plant sources recommended by the International Nomenclature of Cosmetic Ingredients (INCI) database and their effects on the skin (according to the CosIng database).

Species	INCI Name	Description	Functions
*Anchusa* *arvensis*	*Anchusa arvensis* extract	*Anchusa arvensis* extract is the extract of the whole plant.	Skin conditioning
*Arnebia*	*Arnebia euchroma* root extract	*Arnebia euchroma* root extract is the extract of the roots.	Antimicrobial
*Borago officinalis* L.	*Borage* seed oil aminopropanediol amides	*Borage* seed oil aminopropanediol amides is the product obtained by the reaction of Borago officinalis seed oil and aminopropanediol.	Skin conditioning
	*Borage* seed oil peg-8 esters	*Borage* seed oil PEG-8 esters are the product obtained by the transesterification of *Borago officinalis* L., seed oil, and PEG-8.	Skin conditioning, skin conditioning—emollient, surfactant—cleansing, surfactant—emulsifying
	*Borage* seed oil polyglyceryl-4 esters	*Borage* seed oil polyglyceryl-4 esters are the product obtained by the transesterification of Borago officinalis seed oil and polyglycerin-4.	Opacifying, solvent, surfactant—cleansing, surfactant—emulsifying
	*Saccharomyces/Borago officinalis* seed oil/glycerin ferment filtrate	*Saccharomyces/Borago officinalis* seed oil/glycerin ferment filtrate is a filtrate of the product obtained by the fermentation of *Borago officinalis* seed oil and glycerin by the microorganism, saccharomyces.	Skin conditioning—emollient
	*Saccharomyces/Alchemilla vulgaris/Achillea millefolium/Borago officinalis/Eucalyptus globulus/Helichrysum arenarium* ferment extract filtrate	Borago officinalis ferment extract filtrate is a filtrate of the extract of the product obtained by the fermentation of the whole plants, borago officinalis, by the microorganism, saccharomyces.	Antioxidant, skin conditioning
	*Rhizopus/Borago officinalis* seed oil ferment filtrate	*Rhizopus/Borago officinalis* seed oil ferment filtrate is a filtrate of the product obtained by the fermentation of borago officinalis, Boraginaceae, seed oil, by the microorganism rhizopus.	Skin conditioning
	Potassium borageate	Potassium borageate is the potassium salt of the fatty acids derived from *Borago officinalis* seed oil.	Cleansing, surfactant—cleansing
	Peg-9 borageate	Poly(oxy-1,2-ethanediyl), .alpha.-hydro-.omega.-hydroxy-, esters with borage-oil (*Borago officinalis* L. seed) fatty acids (9 mol EO average molar ratio).	Surfactant—emulsifying
	Hydrolyzed *borage* seed oil extract	Hydrolyzed borage seed oil extract is the hydrolysate of the extract of *Borago officinalis* seed oil derived by acid, enzyme, or other method of hydrolysis.	Antioxidant
	Hydrolyzed *borage* seed oil	Hydrolyzed borage seed oil is the hydrolysate of *Borago officinalis* seed oil derived by acid, enzyme, or other method of hydrolysis.	Hair conditioning, skin conditioning
	Dimethiconol borageate	Reaction product of the fatty acids derived from *Borago officinalis* seed oil and poly[oxy(dimethylsilylene), alpha.-hydro, .omega.-hydroxy.	Skin conditioning, skin conditioning—emollient
	*Borago officinalis* leaf water	*Borago officinalis* leaf water is the aqueous solution of the steam distillates obtained from the whole plants.	Anti-sebum, antioxidant, skin conditioning, skin protecting
	*Borago officinalis* seed oil	*Borago officinalis* seed oil is the fixed oil obtained from the seeds.	Skin conditioning, skin conditioning—emollient
	*Borago officinalis* seed extract	*Borago Officinalis* seed extract is an extract of the seeds of *Borago officinalis* L.	Skin conditioning
	*Borago officinalis* leaf extract	*Borago officinalis* leaf extract is the extract of the leaves.	Skin conditioning
	*Borago officinalis* extract	*Borago officinalis* extract is an extract of the herb.	Skin conditioning, skin conditioning—emollient
	*Borago officinalis* ethyl ester	*Borago officinalis* ethyl ester is the ethyl ester of the fatty acids derived from the oil of the seeds.	Skin conditioning
	Borage seed oil/hydrogenated borage seed oil esters	Borage seed oil/hydrogenated borage seed oil esters are the product obtained by the transesterification of Borago.	Skin conditioning—emollient, skin protecting
	Borage seed oil polyglyceryl-6 esters	Borage seed oil polyglyceryl-6 esters are the product obtained by the transesterification of *Borago officinalis* seed oil and polyglyceryl-6.	Skin conditioning, skin conditioning—emollient, surfactant—cleansing, surfactant—emulsifying
*Buglossoides Arvensis* L. *I.M.Johnst.*	*Buglossoides arvensis* seed oil	*Buglossoides arvensis* seed oil is the oil expressed from the seeds.	Skin conditioning, skin conditioning—emollient
*Cordia*	*Cordia salicifolia* extract	*Cordia salicifolia* extract is the extract of the whole plant.	Skin conditioning
	*Cordia obliqua* leaf extract	*Cordia obliqua* leaf extract is an extract of the leaves.	Skin conditioning
	*Cordia curassavica* leaf oil	*Cordia curassavica* leaf oil is the volatile oil obtained from the leaves.	Fragrance
*Echium* L.	*Echium plantagineum* seed oil	*Echium plantagineum* seed oil is the fixed oil obtained from the seeds.	Skin conditioning, solvent
	*Echium lycopsis* root extract	*Echium lycopsis* root extract is an extract of the roots.	Skin conditioning
	*Echium lycopsis* fruit oil	*Echium lycopsis* fruit oil is the oil expressed from the fruit.	Skin conditioning
*Lappula*	*Lappula squarrosa* seed oil	*Lappula squarrosa* seed oil is the oil expressed from the seeds of *Lappula squarrosa.*	Skin conditioning, skin protecting
*Lithospermum erythrorhizon*	*Lithospermum erythrorhizon* root	*Lithospermum Erythrorhizon* root is the powdered root of *Lithospermum erythrorhizon*.	Skin conditioning
	*Lithospermum erythrorhizon* root oil ferment filtrate	*Lithospermum erythrorhizon* root oil ferment filtrate is a filtrate of the product obtained by the fermentation of *Lithospermum erythrorhizon* root oil by the microorganism saccharomyces.	Skin conditioning
	*Lithospermum erythrorhizon* root ferment filtrate extract	*Lithospermum erythrorhizon* root ferment extract filtrate is a filtrate of the extract of product obtained by the fermentation by the microorganism, *Pseudozyma epicola*.	Skin conditioning, skin conditioning—emollient, emulsion stabilizing, hair conditioning, humectant, skin conditioning, skin protecting
	*Lithospermum* root extract serum succinate albumin	*Lithospermum* root extract serum albumin succinate is the product obtained by the reaction of *Lithospermum erythrorhizon* root extract with succinylated serum albumin.	Skin conditioning
*Lithospermum officinale* L.	*Lithospermum officinale* extract	*Lithospermum officinale* extract is an extract of the whole plant of the gromwell.	Fragrance, skin protecting
	*Lithospermum officinale* root extract	*Lithospermum officinale* root extract is an extract of the roots of the gromwell.	Skin conditioning
	*Lithospermum officinale* seed oil	*Lithospermum officinale* seed oil is the oil expressed from the seeds of the gromwell.	Skin conditioning
*Mertensis Maritima*	*Mertensia maritima* extract	*Mertensia maritima* extract is the extract of the whole plant.	Skin conditioning
*Pulmonaria officinalis* L.	*Pulmonaria officinalis* extract	*Pulmonaria officinalis* extract is an extract of the whole plant of the lungwort.	Astringent, skin conditioning, skin conditioning—emollient
*Symphytum officinale* L.	*Symphytum officinale* root extract	*Symphytum officinale* root extract is the extract of the roots of the comfrey.	Anti-seborrheic, skin conditioning, soothing
	*Symphytum officinale* root cell extract	*Symphytum officinale* root cell extract is the extract of a culture of the root cells of the comfrey.	Skin conditioning
	*Symphytum officinale* rhizome/root extract	*Symphytum officinale* rhizome/Root extract is the extract of the rhizomes and roots of *Symphytum officinale*.	Skin conditioning
	*Symphytum officinale* leaf powder	*Symphytum officinale* leaf powder is a powder of finely ground leaves from the comfrey.	Abrasive
	*Symphytum officinale* leaf extract	*Symphytum officinale* leaf extract is an extract of the leaves of the comfrey.	Skin conditioning
	*Symphytum officinale* extract	*Symphytum officinale* extract is the extract of the whole plant.	Skin conditioning—miscellaneous
	*Symphytum officinale* callus culture lysate	*Symphytum officinale* callus culture lysate is a lysate of a suspension of the cultured callus cells.	Skin conditioning
	*Symphytum officinale* callus culture extract	*Symphytum officinale* callus culture extract is the extract of a culture of the callus.	Skin conditioning
*Trichodesma zeylanicum*	*Trichodesma zeylanicum* oil	*Trichodesma zeylanicum* oil is the fixed oil obtained from the plant.	Skin conditioning, skin conditioning—emollient

## Data Availability

No new data were created or analyzed in this study.
